# Defining early carcinogenic markers for the prediction of the hazard potential of mineral fibres in an in vitro human 3D bronchial model

**DOI:** 10.1007/s00204-026-04471-3

**Published:** 2026-06-15

**Authors:** Vanessa Almonti, Serena Mirata, Mario Passalacqua, Stefania Vernazza, Sara Tirendi, Sara Ferrando, Beatrice Risso, Elena Grasselli, Giulia De Negri Atanasio, Anna Maria Bassi, Alessandro F. Gualtieri, Sonia Scarfì

**Affiliations:** 1 Wielkopolska Center for Advanced Technologies, Adam Mickiewicz University, 61-614 Poznan, Poland; 2 Department of Earth, Environment and Life Sciences, University of Genova, 16132 Genova, Italy; 3 Inter-University Center for the Promotion of the 3Rs Principles in Teaching & Research (Centro 3R), 10125 Torino, Italy; 4 Department of Experimental Medicine, University of Genova, 16132 Genova, Italy; 5 National Biodiversity Future Center (NBFC), 90133 Palermo, Italy; 6 National Center for the Development of New Technologies in Agriculture (Agritech), Napoli, Italy; 7 Istituto Nazionale Biostrutture e Biosistemi (INBB), 00136 Roma, Italy; 8 Department of Chemical and Geological Sciences, University of Modena and Reggio Emilia, 41125 Modena, Italy

**Keywords:** Mineral fibres, 3D lung model, EpiAirway™, Key characteristics of carcinogens

## Abstract

This study aimed to identify early carcinogenic markers to predict the hazard potential of mineral fibres using a human 3D bronchial model. Traditional 2D cell models inadequately mimic lung complexity, prompting the use of more physiologically relevant 3D systems. We investigated the effects of crocidolite (CRO) and two size fractions of chrysotile (≤ and > 5 μm length: CHR S and CHR L) to elucidate early toxicological mechanisms and relate them to the IARC key characteristics (KCs) of carcinogens. Initial analyses (MTT, TEER, and histology) at 24–48 h showed a transient acute toxicity and tissue resilience, indicating limited predictive value at this time point. However, after 12 d of exposure, fibre-treated tissues exhibited persistent alterations corresponding to established IARC KCs, suggesting the early onset of cell transformation pathways. Key findings included: sustained inflammatory signalling, characterized by prolonged overexpression of IL-1β, IL-6, and IL-8; ii) persistent genotoxicity, marked by the prolonged nuclear positivity of γH2AX, indicating DNA double-strand breaks without significant cell death; and iii) induction of fibrotic and epithelial-to-mesenchymal transition (EMT) signals, including the increased expression of key markers such as TGF-β, VEGF, ZEB-2, N-Cadherin, Vimentin, and Mesothelin. These alterations correspond to IARC KCs 2, 6, and 8. They were observed within a short time frame of tissue treatment and could serve as reliable, early predictive endpoints for in vitro toxicological tests. We propose that integrating these specific KC-based biosignatures in 3D lung models with physicochemical fibre characterization may provide a robust framework for predictive assessment of inhalable fibre carcinogenicity.

**Supplementary Information:**

The online version contains supplementary material available at https://doi.org/10.1007/s00204-026-04471-3.

## Introduction

Traditionally, inhalation toxicology investigations have relied heavily on in vivo testing methodologies to establish the toxicity profiles of airborne pollutants. Manufacturers and regulatory agencies employ in vivo rodent acute inhalation toxicity tests (OECD 2009a, b, 2018), which address direct impacts on the respiratory system (i.e., local respiratory effects) as well as systemic effects on other organs (Wallace et al. 2023). However, due to increasing animal welfare concerns and significant species-specific differences, the need for more predictive human data on inhalation toxicity has grown. In this regard, several past studies using rodent models have demonstrated limited relevance to human physiology (Fröhlich and Salar-Behzadi 2014; Movia et al. 2020). As a result, human-relevant in vitro models have emerged as promising platforms for inhalation toxicity assessments. Although several advanced in vitro approaches, including immortalised co-culture systems with epithelial, endothelial, and immune components, have significantly improved physiological relevance (Costa et al. 2019; Mitchell et al. 2024), fully differentiated human airway epithelial tissues cultured at the air–liquid interface (ALI) offer complementary advantages by reproducing mucociliary differentiation and barrier integrity of the tracheobronchial region. Over the past decade, different three-dimensional (3D) airway tissue models, going from microspheres to epithelial barrier reconstructions, have been increasingly employed in toxicological studies and safety assessment of various substances, including multi-walled carbon nanotubes, nanomaterials, chemical compounds, drugs and e-cigarette smoke (Neilson et al. 2015; Jackson et al. 2018; Kabadi et al. 2019; Lehner et al. 2022; Sharma et al. 2023).

Asbestos is a well-known environmental contaminant inducing serious effects on human health, leading to clinical manifestations ranging from chronic lung inflammation, asbestosis, and fibrotic lung disease to malignancies, such as lung cancer and malignant pleural mesothelioma (MPM) (Attanoos 2010). Indeed, amphibole asbestos (including actinolite, amosite, anthophyllite, crocidolite, and tremolite), along with chrysotile (the only type of asbestos belonging to the serpentine group) were included in *Group 1* carcinogens by the International Agency for Research on Cancer (IARC 2012). However, the hazard of chrysotile, which accounts for 95% of the asbestos marketed in the last century, has often been questioned by mine industries and by associations funded by asbestos mining companies in Russia, Kazakhstan and Zimbabwe (Baur and Frank 2021). In this regard, the low biodurability of chrysotile supports the assumption that its fibres have a lower toxic/carcinogenic potential compared to amphibole asbestos. This controversial view is further supported by the “amphibole hypothesis”, which attributes chrysotile potential toxicity entirely to the presence of amphibole asbestos contamination rather than to the pure mineral itself (Stayner et al. 1996). As a result, chrysotile continues to be widely exploited and marketed in numerous countries worldwide, by claiming a “safe use” (Gualtieri 2021 and references herein).

Morphometric parameters of asbestos fibres (e.g., length, width, aspect ratio) are considered important factors for toxicity risk assessment in directly exposed tissues, even though the correlation between the fibre size and their harmful effects remains debated. In this regard, Gualtieri et al. (2023) reported that even the shortest fibres (≤ 5 μm in length and 0.25 μm in width) should be included when evaluating the so-called “asbestos issue”. Several studies have linked fibre dimensions to tumorigenesis, showing that long (length > 5 μm), thin (diameter ≤ 0.5 μm) fibres exhibit greater carcinogenic potential than short fibres (Donaldson et al. 2010; Aust et al. 2011). At the same time, some authors pointed out the high content of short fibres in mesothelioma patients; for instance, Suzuki et al. (2005) found that most fibres in affected tissues were ≤ 5 μm long and 0.25 μm wide.

After reaching the lower respiratory tract, the inhaled fibres interact with resident alveolar macrophages that mediate their removal *via* phagocytosis and initiate the inflammatory process; conversely, alveolar epithelial cells act as a physical barrier protecting the underlying layers of fibroblasts and endothelial cells from fibre penetration (Barlow and Mossman 2025). In this context, 3D human bronchial models can provide an advanced platform to investigate the sustained molecular detrimental effects of mineral fibres, as they better reproduce direct and prolonged exposure of the upper airway epithelium. The initiation of chronic inflammatory processes (i.e., fibrosis) and epithelial-to-mesenchymal transition (EMT) are recognised mechanisms involved in the early stages of asbestos-related carcinogenesis (Matsuzaki et al. 2012; Gulino et al. 2016). Specifically, investigating these processes in human-relevant in vitro airway models may contribute to the identification of the early carcinogenic markers triggered by exposure to hazardous inhalable fibres, ultimately improving the current predictive frameworks assessing their effect on human health.

Taking advantage of the tracheobronchial epithelial tissue model EpiAirway™, which reproduces the first cellular layers of the lung epithelial barrier, including ciliated and mucus-secreting cells (Sanchez-Guzman et al. 2021), the present study aimed to analyse the effects of two different size fractions (i.e., ≤ and > 5 μm length) of commercial chrysotile fibres from the Orenburg region (Scognamiglio et al. 2021), with crocidolite employed as a positive control. While previous in vitro studies investigated asbestos toxicity in 2D or co-culture models, the present work describes a long-term exposure approach (i.e. up to 12 d of treatment) based on a 3D lung respiratory tissue. This strategy enables the evaluation of asbestos-induced toxicity under more physiologically relevant conditions and contributes to the identification of early prognostic markers of carcinogenicity selected among the 10 IARC key characteristics (KC) of carcinogens (Smith et al. 2016). Through this research, we aim to gain insight into the effects of inhaled mineral fibres and pave the way for the development of more effective safety assessment strategies.

## Methods

### Chemicals

All reagents were acquired from SIGMA-ALDRICH (Milan, Italy), unless otherwise stated.

### Mineral fibres

**Fig. 1:**
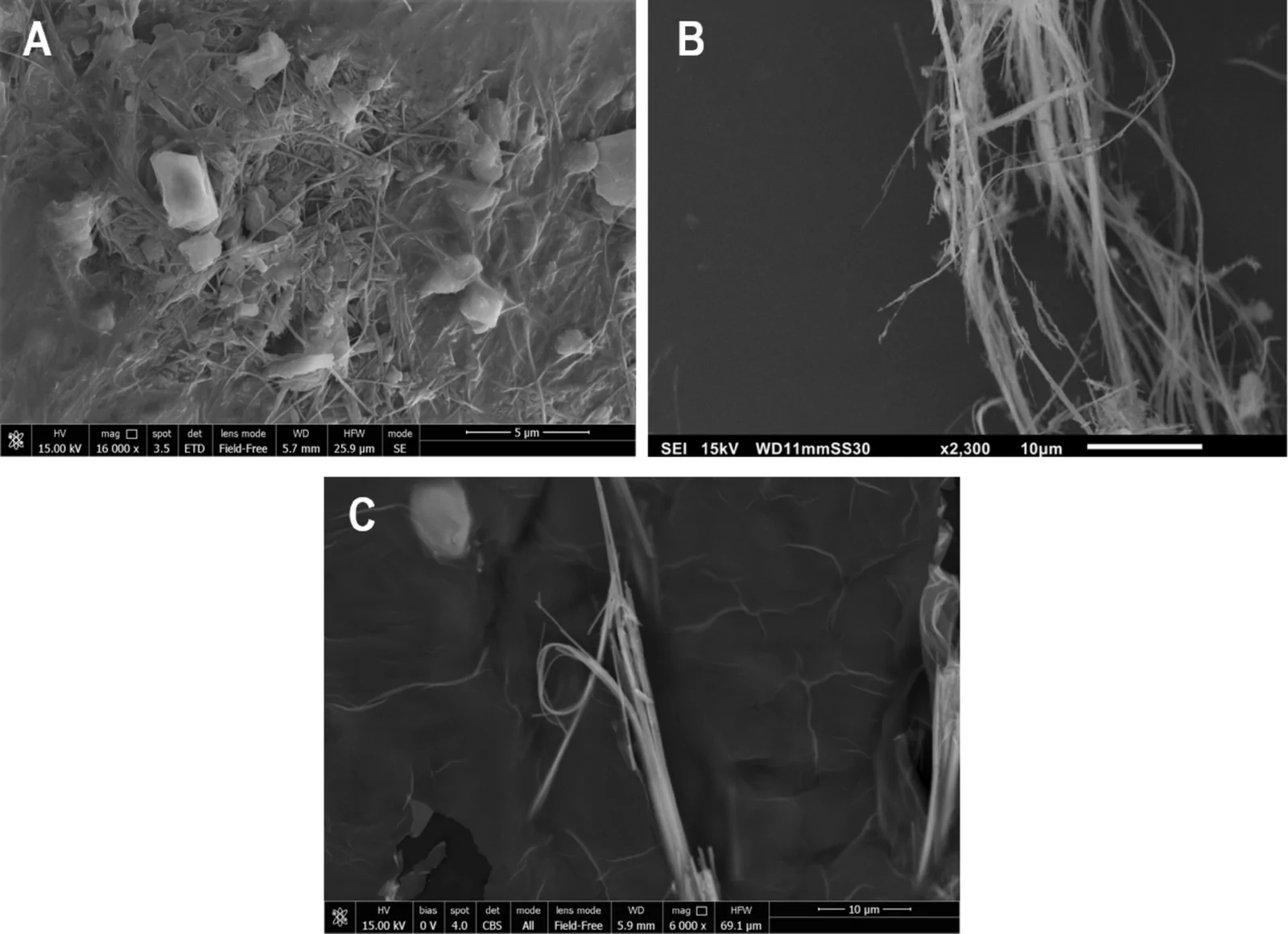
SEM images of the mineral fibres used for the experiments. **A** chrysotile (Southern Ural Mountains) after 40 min of milling (CHR S); **B** the same chrysotile after 5 min of milling (CHR L); **C** UICC crocidolite standard (South Africa)

The mineral fibres used in this study were:

- Chrysotile from Orenburg Minerals mine (Southern Ural Mountains, Russia), which has been accurately described in Di Giuseppe et al. (2021). To prepare the two different size fractions, namely a shorter fraction (L ≤ 5 μm) and a longer fraction (L > 5 μm), chrysotile fibres were subjected to cryogenic milling as reported by Scognamiglio et al. (2021). Their morphometric characteristics are reported in Table 1, while representative images are reported in Fig. 1A–B.
- UICC crocidolite standard (South African, NB #4173-111-3), which has been described by Gualtieri et al. (2018), was used as a positive carcinogenic control (Fig. 1C).

**Table 1:** Summary of the morphometric characteristics of CHR S, CHR L, and CRO

Mineral fibre	Length (µm)	Width (µm)	SSA (m^2^/g)	Impurities	References
CHR S	1.91(0.21)	0.15(0.10)	30.0	Lizardite-1T, magnetite, hydromagnesite, calcite	Di Giuseppe et al. (2021) Scognamiglio et al. (2021)
CHR L	29.8(3.08)	0.4(0.14)	28.9	Lizardite-1T, magnetite, hydromagnesite, calcite	Di Giuseppe et al. (2021) Scognamiglio et al. (2021)
CRO	25.0(9.9)	0.3(0.13)	16.1	Hematite, magnetite, quartz	Gualtieri et al. (2018)

Mineral fibres were sterilised by autoclave standard treatment (121 °C, 20 min), which preserves their morphology and crystalline structure (IARC 2012). To prepare fibre stock suspensions, 5 mg of sterilised fibres were suspended in 1 ml of phosphate buffered saline (PBS) and tip sonicated using a UP50H Ultrasonic Processor (Hielscher Ultrasonics GmbH, Teltow, Germany) for 5 min in sterile conditions. Stock suspensions were stored at − 20 °C and, on the day of the experiments, they were thawed and diluted to the desired concentration.

### The EpiAirway™ model

The human mucociliary epithelial tissue models (EpiAirway™) consisting of primary Normal Human Bronchial Epithelial cells (NHBE) from multiple donors were purchased from MatTek Corporation (Ashland, MA). Upon arrival, they were placed into fresh EpiAirway™ culture medium and incubated (37 °C, 5% CO_2_) under ALI conditions, with cell culture medium supplied from the basolateral side. After overnight incubation, the apical surface of the EpiAirway™ models was rinsed twice with PBS to remove residual liquid and mucus before exposure to mineral fibres. Fibre suspensions were prepared at 100–200 µg/mL (final concentrations) in 100 µL of PBS and were directly applied to the apical surface of the tissues for 24 h, 48 h, or 12 days. For all experiments, results were normalised respect to a vehicle control consisting of PBS alone (UT).

### MTT analyses

After exposure to the different mineral fibres, the tissues viability was evaluated according to the EpiAirway™ Toxicity Testing Protocol. Briefly, tissues were exposed to 100–200 µg/ml of CRO, CHR S, and CHR L for 24 and 48 h. For the 12 day-treatment, tissues were exposed to 100 µg/ml mineral fibres with either single (day 0) or repeated (day 0 and day 6) administrations. Experiments were performed in three independent biological replicates, each with technical duplicates. At the end of the incubation (24, 48 h and 12 d), the tissues were placed into a 24-well plate containing 300 µL of pre-warmed MTT reagent, and incubated at 37 °C with 5% CO_2_ for 90 min. After incubation, each tissue insert was gently blotted to remove the remaining MTT reagent, immersed in 2 ml of isopropanol, and placed on an orbital shaker at room temperature in the dark for 2 h. Then, formazan absorbance was read at 570 nm with a plate reader (BMG Labtech, Ortenberg, Germany).

### Trans-epithelial electrical resistance (TEER) measurement

The integrity of the cell monolayer was monitored by measuring the trans-epithelial electrical resistance (TEER) with an epithelial voltmeter (EVOM, Merck Millipore) every other day until day 12 of the single or double fibre treatment. Before the quantification, the electrodes were sterilized in ethanol solution (70% v/v) and then equilibrated with TEER solution (MatTek Corporation) for 3 min. The tissues were transferred to a 12-well and left to equilibrate for 3 min in PBS before TEER measurement with the electrodes. The computed values were obtained by subtracting the resistance values of the insert membrane in TEER solution and multiplying them for the surface area of the respective membrane. For each condition, measurements were taken every other day before changing the culture medium.

### Histological staining

After exposure to 100 µg/mL of asbestos fibres for 48 h and 12 d, EpiAirway™ tissues were fixed for 1 h in 4% paraformaldehyde in PBS, rinsed for 1 h in three changes of PBS, dehydrated through a graded ethanol series, and, after removal of the plastic insert, cleared in BioClear (Bio-Optica, Milano, Italy), and embedded in paraffin (Paraplast Plus, Sigma Aldrich). Paraffin blocks were sectioned at 5 μm thickness using a Reichert-Jung microtome (Germany). Sections were stained using Haematoxylin and Eosin (H&E) (Bio-Optica, Milano, Italy), Alcian Blue - Periodic Acid-Schiff (AB-PAS) and Sirius Red (Sigma Aldrich). After staining, sections were dehydrated through an ethanol series, cleared in Bioclear, and mounted with Eukitt mounting medium (ORSAtec GmbH, Germany) and coverslips. Histological slides were examined using a transmitted light microscope Leica DMRB equipped with polarized light and with a Moticam 3+ (Motic Europe, Barcelona, Spain) or through a transmitted light microscopy Olympus BX60 equipped with a Microvisioneer (Esslingen am Neckar, Germany) camera and acquisition software.

### Evaluation of the inflammatory potential

EpiAirway™ tissues were exposed to 100 µg/ml (final concentration) mineral fibres for 24 h, 48 h and 12 d before evaluating the gene expression profile of several inflammatory mediators (i.e., IL-1β, IL-6, IL-8, MCP-1, and TNF-α) by qPCR. Moreover, a positive inflammatory control with LPS (5 µg/ml final concentration) was prepared as well for short-term experiments. Additionally, the gene expression of growth factors (VEGF and TGF-β), fibrotic mediators (Fibronectin, α-SMA, Collagen-1 A), anti-inflammatory cytokines (IL-10), and EMT markers (N-Cadherin, E-Cadherin, TWIST-1, ZEB-2, and Vimentin) were also measured in the tissues after 12 d of fibre treatment. Values were normalized to HPRT-1 mRNA expression, which was chosen after an initial screening of different housekeeping genes (i.e., β-actin, GAPDH, HPRT-1, and Ubiquitin). RNA extraction, cDNA retrotranscription, and qPCR analyses were performed as described in Giordani et al. (2025). All primer pairs are listed in Table 2.

**Table 2:** List of primer pairs used in the qPCR analyses

Gene	GenBank (a.*n*.)	Forward	Reverse	Product size (bp)
IL-1β	NM_000576.3	TGATGGCTTATTACAGTGGCAATG	GTAGTGGTGGTCGGAGATTCG	140
IL-6	NM_001318095.2	CAGATTTGAGAGTAGTGAGGAAC	CGCAGAATGAGATGAGTTGTC	194
IL-8	NM_000584.4	AATTCATTCTCTGTGGTATC	CCAGGAATCTTGTATTGC	127
MCP-1	NM_002982	CTTCTGTGCCTGCTGCTC	CTTGCTGCTGGTGATTCTTC	156
TNF-α	NM_000594.4	GTGAGGAGGACGAACATC	GAGCCAGAAGAGGTTGAG	113
TGF-β2	NM_001135599.3	AACCTCTAACCATTCTCTACTACA	CGTCGTCATCATCATTATCATCA	149
VEGF	NM_001025370.1	TTCGGGCTGTTCTCGCTTC	CTCTCCTCTTCCTTCTCTTCTTCC	142
N-CADH	NM_001792.5	GGTGGAGGAGAAGAAGACCAG	GGCATCAGGCTCCACAGT	72
E-CADH	NM_004360.5	AGACAGAGCGGAACTATG	TCAATCATCCTCAGCATCA	95
TWIST1	NM_000474.4	CGGCCAGGTACATCGACT	CATCTTGGAGTCCAGCTCGT	62
ZEB2	NM_014795.4	CAAGAGGCGCAAACAAGC	AACCTGTGTCCACTACATTGTCA	71
FIBRO	NM_002026	CCAGCAGAGGCATAAGGTTC	GGTCAAAGCACGAGTCATCC	95
α-SMA	NM_001141945.1	TTGAGAAGAGTTACGAGTTG	GGACATTGTTAGCATAGAGG	189
COL-1 A	NM_000088.3	CTTTGCATTCATCTCTCAAACTTAGTTTT	CCCCGCATGGGTCTTCA	163
VIMENTIN	NM_003380.5	ACCAAGACCTGCTCAATGTTAAGA	CCTGCTCTCCTCGCCTTC	83
HPRT-1	NM_000194.3	GGTCAGGCAGTATAATCCAAAG	TTCATTATAGTCAAGGGCATATCC	144

The activation of pro-inflammatory pathways was also evaluated by measuring the extracellular release of IL-1β, IL-6, IL-8, and TNF-α. At the end of the experimental time (24 and 48 h), the culture media were collected, centrifuged at 360 RCF for 10 min and stored at −20 °C. Then, cytokine and chemokine concentrations in the culture medium were quantified using the MILLIPLEX^®^ Human Cytokine/Chemokine/Growth Factor Panel A (Merck KGaA, Darmstadt, Germany), following the manufacturer’s instructions. Raw values were normalized to the MTT-derived cytotoxic index to account for differences in tissue viability at the time of collection.

### Protein expression analyses

After 24, 48 h, and 12 d of single-dose fibre treatments, whole tissue lysates were collected in 180 µL of RIPA lysis buffer (Sigma Aldrich) plus a protease inhibitor cocktail (Roche Diagnostic GmbH, Mannheim, Germany). Samples were sonicated in ice to ensure complete tissue disruption and protein solubilization, and then centrifuged at 14,000 × g for 15 min at 4 °C. The supernatant was collected and stored at − 80 °C. Protein content was determined by Bradford assay (Bio-Rad Laboratories srl, Milan, Italy). Then 20 µg of proteins were resolved on NuPAGE™ 4 to 12%, Bis-Tris Midi Protein Gels (Life Technologies, Milan, Italy) in NuPAGE™ MES SDS Running Buffer (1X) and transferred onto SureLock™ Tandem Midi Pre-cut Membranes and Filters nitrocellulose (Life Technologies). After the blotting transfer step, the membrane was labelled with 10 ml of No-Stain™ Membrane Labelling (Life Technologies) according to the manufacturer’s instructions. Membranes were imaged using the iBright Imaging System (Life Technologies) with 455–485 nm excitation and 565–615 nm emission. The TPN signal was quantified using iBright™ Analysis Software and used to normalize all subsequent chemiluminescent signals, providing a fold-change relative to untreated control. Membrane from early time points (24 and 48 h) were probed with specific primary antibodies against rabbit anti-phospho-NF-kB p65 (Ser 536) (clone 93H1, #3033), rabbit anti-PARP 89/116 (#9542) (Cell Signalling Technology), rabbit anti-H2AX (#ab11175) (Abcam), mouse anti-γH2AX (phospho-Ser139) (clone 9F3, #ab26350) (Abcam), followed by incubation with HRP-conjugated secondary antibodies (NA9340V and NA931V, against rabbit and mouse primary antibodies, respectively, Amersham Life Science, Milan, Italy). In addition, the 12-day treatment membranes were also probed with rabbit anti-Mesothelin (D9R5G, #99966), rabbit anti-β-Catenin (D10A8 #8480), rabbit anti-Claudin-1 (D5H1D #13255), and rabbit anti-ZO-1 (D7D12 #8193) (Cell Signalling Technology). Proteins were detected by SuperSignal™ West Pico PLUS Chemiluminescent Substrate (Thermo Fisher Scientific, MA, USA), imaged with the iBright system. All signals were normalized to the total protein signal and expressed as fold change relative to untreated controls.

### Statistical analysis

Statistical analysis was performed with GraphPad Prism 9 (GraphPad Software, Inc., La Jolla, CA, USA). Statistical significance was assessed by One-way ANOVA or Two-way ANOVA, followed by Bonferroni post-test. A *P*-value ≤ 0.05 was defined as statistically significant.

## Results

### MTT cytotoxicity index

**Fig. 2:**
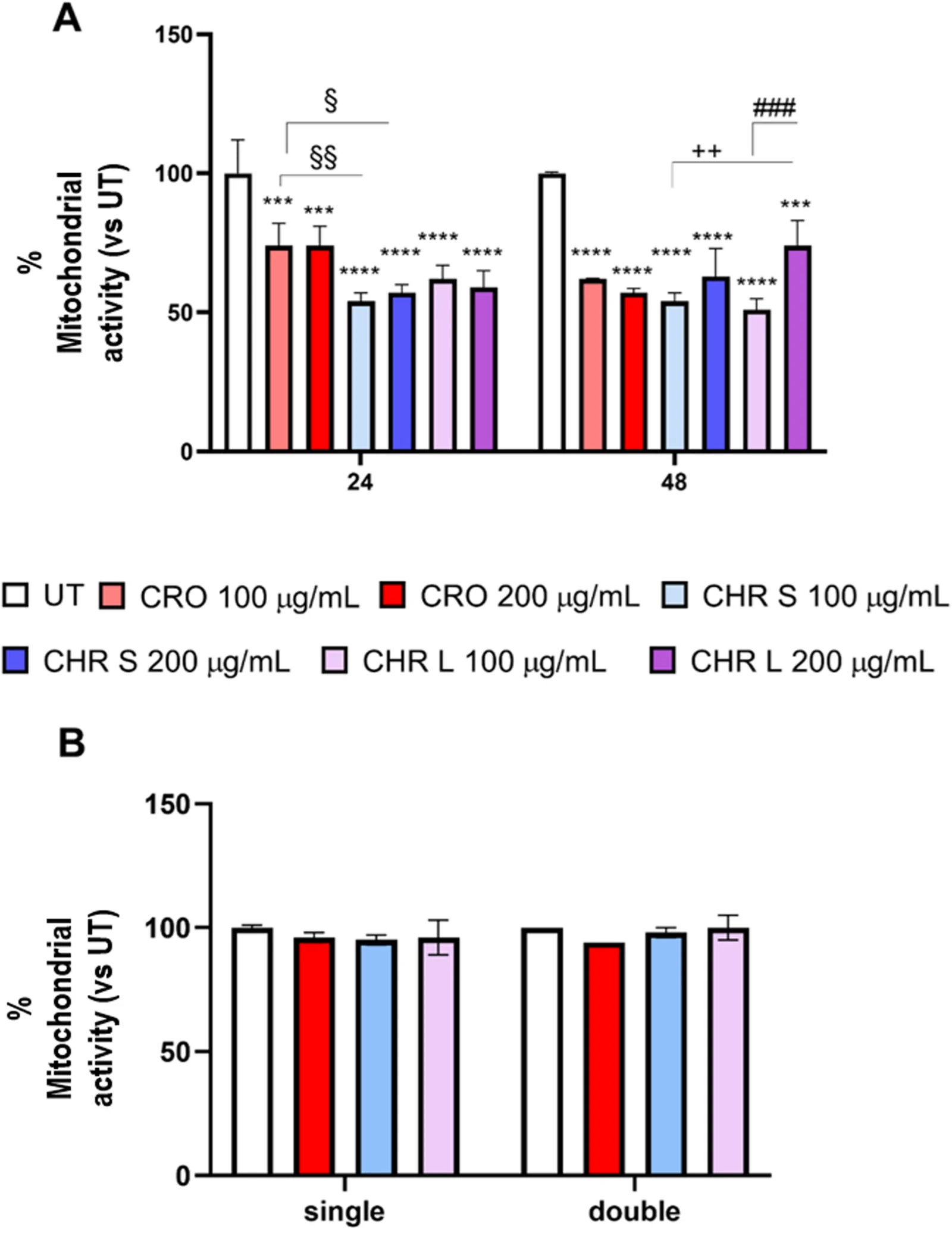
Evaluation of tissue mitochondrial activity by MTT test. **A** Effects of CHR S and CHR L (CRO used as positive control) at different concentrations (100 and 200 µg/ml) after 24 and 48 h of exposure. **B** Effects of CHR S and CHR L (CRO used as positive control) at 100 µg/ml after 12 days with one or double exposure. The second exposure was repeated after six days. Data are expressed as mean ± SD of three independent biological experiments, each performed in duplicate. Statistical analysis was performed using two-way ANOVA (treatment x time) followed by Bonferroni’s post-test. Comparisons were made between each treatment and the corresponding UT control at the same time point. ***p < 0.001, **p < 0.01 vs. UT

The MTT assay revealed changes in tissue metabolic activity following exposure to crocidolite (CRO), short chrysotile (CHR S), and long chrysotile (CHR L) over 24–48 h (Fig. 2A) and 12 d (Fig. 2B). At 24 h, CRO induced approximately 25% cytotoxicity compared to vehicle controls at both concentrations tested, whereas CHR S and CHR L showed higher cytotoxic effects (46% and 38% at 100 µg/mL, and 43% and 41% at 200 µg/mL, respectively). After 48 h, CRO further reduced metabolic activity (38% and 43% at 100 and 200 µg/mL, respectively), whereas both CHR fractions did not show additional decreases and instead displayed a slight recovery of mitochondrial activity, possibly indicating a compensatory metabolic response of the surviving cells.

In contrast, the tissue toxicity assessed after 12 d of exposure to a single or double fibre dose showed no significant variation compared to the untreated tissues (Fig. 2B), suggesting a recovery of the epithelial tissue after the initial damage induced by the fibres in the first 48 h.

**Fig. 3:**
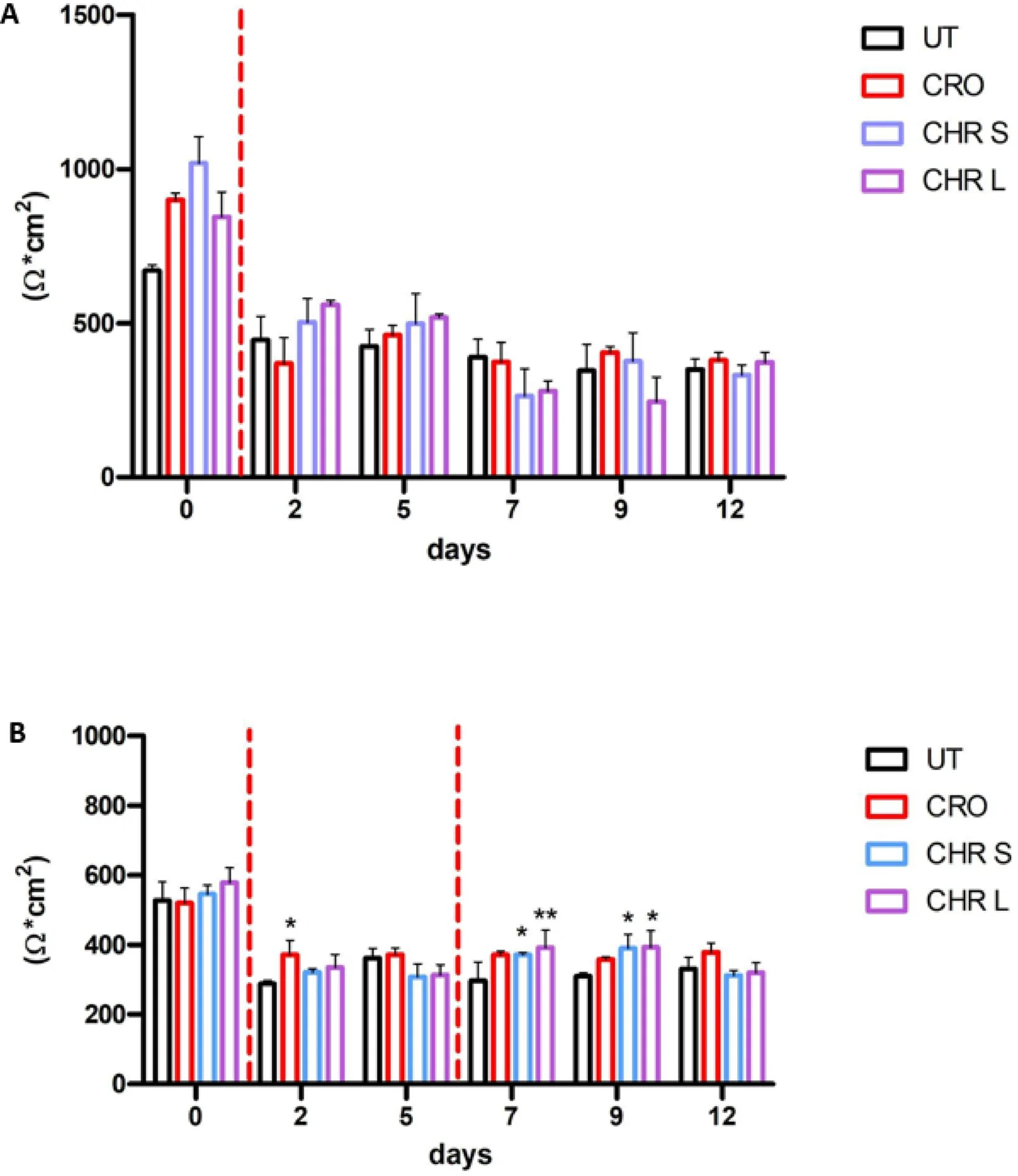
**A** TEER assessment in EpiAirway™ tissues cultured in air-liquid interface (ALI) condition for 12 days following fibre exposure (100 µg/mL). At day 0, the TEER was read before exposure to CHR S, CHR L, and CRO mineral fibres (100 µg/ml). **B** TEER following double-dose exposure administered at day 0 and day 6. Dotted red lines indicate fibre administration. Values represent the mean ± SD of 3 separated experiments run in duplicate. *p < 0.05, **p < 0.01 vs. UT (Two-way ANOVA followed by Bonferroni post-test)

### Trans-epithelial electrical resistance (TEER) measurement

TEER is considered an index of the integrity of the organism cell barriers and epithelia, calculated as the resistance that the whole barrier (tissue plus basal membrane) opposes to the passage of an electric current. Variations in TEER values may result from multiple factors such as temperature, culture medium formulation, passage number of the cells in culture, and integrity of the whole tissue (Karakocak et al. 2023). Figure 3 shows TEER measurements of EpiAirway™ tissues over a 12-day period following either a single (panel **A**) or double (panel **B**) asbestos exposure. At baseline (day 0), before fibre treatments, all TEER values ranged between 500 and 1000 Ω × cm^2^. After 2 days of treatment, all values decreased significantly in both treated and untreated tissues under single- and double-exposure conditions. In the single-dose setting (panel A) the highest drop at day 2 was observed in CRO- and CHR S-treated tissues (approximately 50% compared to day 0), whereas CHR L-treated and control tissues showed a smaller decrease (about 20%). Thereafter, TEER values slightly decreased in all treatments and in control tissues homogeneously, with no significant differences among them up to day 12. Similarly, in the double-exposure experiment (panel **B**), TEER values decreased by approximately 35% at day 2 in both untreated and fibre-treated tissues, reaching values between 300 and 400 Ω × cm². In the following days, the values, albeit with some fluctuations, remained within a comparable range across all conditions until the end of the monitoring period. In conclusion, neither single nor double fibre exposure induced significant TEER alterations compared to controls by day 12. Considering these findings and the MTT results at 12 d, all subsequent experiments were performed using the single-dose protocol, as no significant differences could be observed between the two exposure settings.

### Histological analyses of EpiAirway™ tissues

**Fig. 4:**
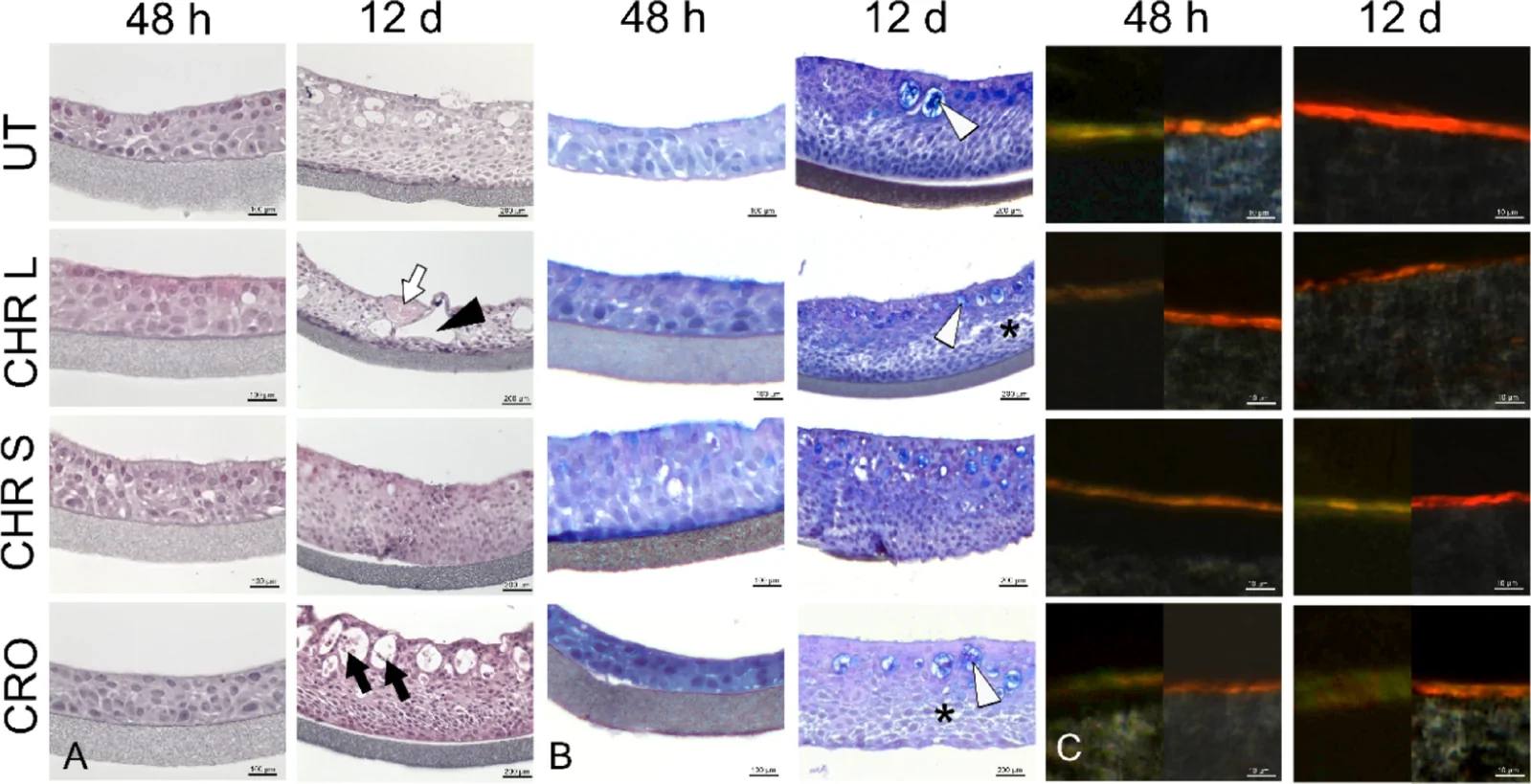
Histological analysis of the EpiAirway™ mucociliary epithelium following asbestos fibre exposure. The cutting plane is perpendicular to the microporous membrane (the bottom grey layer) on which the tissue was cultured. **A** Representative sections stained with Haematoxylin-Eosin (H&E) to evaluate general tissue morphology and epithelial integrity at 48 h and 12 d post-treatment with CHR S, CHR L, or CRO, the latter used as positive control. White arrow: large intercellular spaces with weakly eosinophilic acellular material. Arrowhead: large intercellular empty spaces. Arrows: inclusions in the mucus cells. Scale bar: 100 μm at 48 h, 200 μm at 12 d, respectively. **B** Representative sections stained with Alcian Blue-PAS to visualise mucins and glycosaminoglycans within the bronchial epithelium at 48 h and at 12 d post treatment. White arrowheads: mucous cells. Asterisks: spongiosis. Scale bar: 100 μm at 48 h, 200 μm at 12 d, respectively. **C** Representative sections stained with Sirius Red. Under polarized light, thin collagen fibres appear green, intermediate fibres yellow/orange, and thick mature fibres red. Sections were analysed at 48 h and at 12 d post treatment. Scale bar: 10 μm

The morphology of the EpiAirway™ tissues was investigated through Haematoxylin and Eosin (H&E), Alcian-Blue PAS and Sirius Red tissue staining, in order to highlight the tissue structure, the mucopolysaccharide, and collagen deposition of the bronchial epithelium, respectively (Fig. 4). Untreated epithelia (UT) were approximately 4–5 cell layers thick after 48 h and lacked mucous cells. After 12 days, the thickness had more than doubled (to approximately 12 cell layers), and numerous mucous cells were present, containing a secretion both alcianophilic and PAS-positive.

No treatment induced histological alterations after 48 h. The CHR-S treatment did not show any evident differences even after 12 d. Epithelia treated with CHR-L for 12 d exhibited spongiosis and the presence of large intercellular spaces, appearing either empty or filled with weakly eosinophilic acellular material. The CRO treatment resulted in abundant mucous cells, Alcian–PAS positive, with inclusions that were also visible in haematoxylin–eosin staining (Fig. 4A, B).

In Sirius red staining (Fig. 4C), which highlights collagen fibre organization, control tissues (UT) at 48 h contained both thick (mature) and thin protein fibrils showing a differential staining pattern consistent with variations in collagen fibre maturation (green staining: thin fibrils; yellow/orange staining: intermediate; red staining: thick collagen fibrils). Instead, at 12 d, control tissues showed predominantly thick, mature collagen fibres. At 48 h, fibre-treated EpiAirway™ tissues mostly displayed mature collagen fibrils (prevalence of red staining), except for CRO-treated tissues, which also showed the presence of thin, newly synthesized fibrils (green staining). At 12 d, CHR L-treated tissues predominantly exhibited thick, mature collagen fibrils, whereas CHR S and, more markedly, CRO-treated tissues displayed a mixed pattern of thin and thick collagen fibres, indicating both newly synthesized and mature collagen deposits in asbestos exposed tissues.

**Fig. 5:**
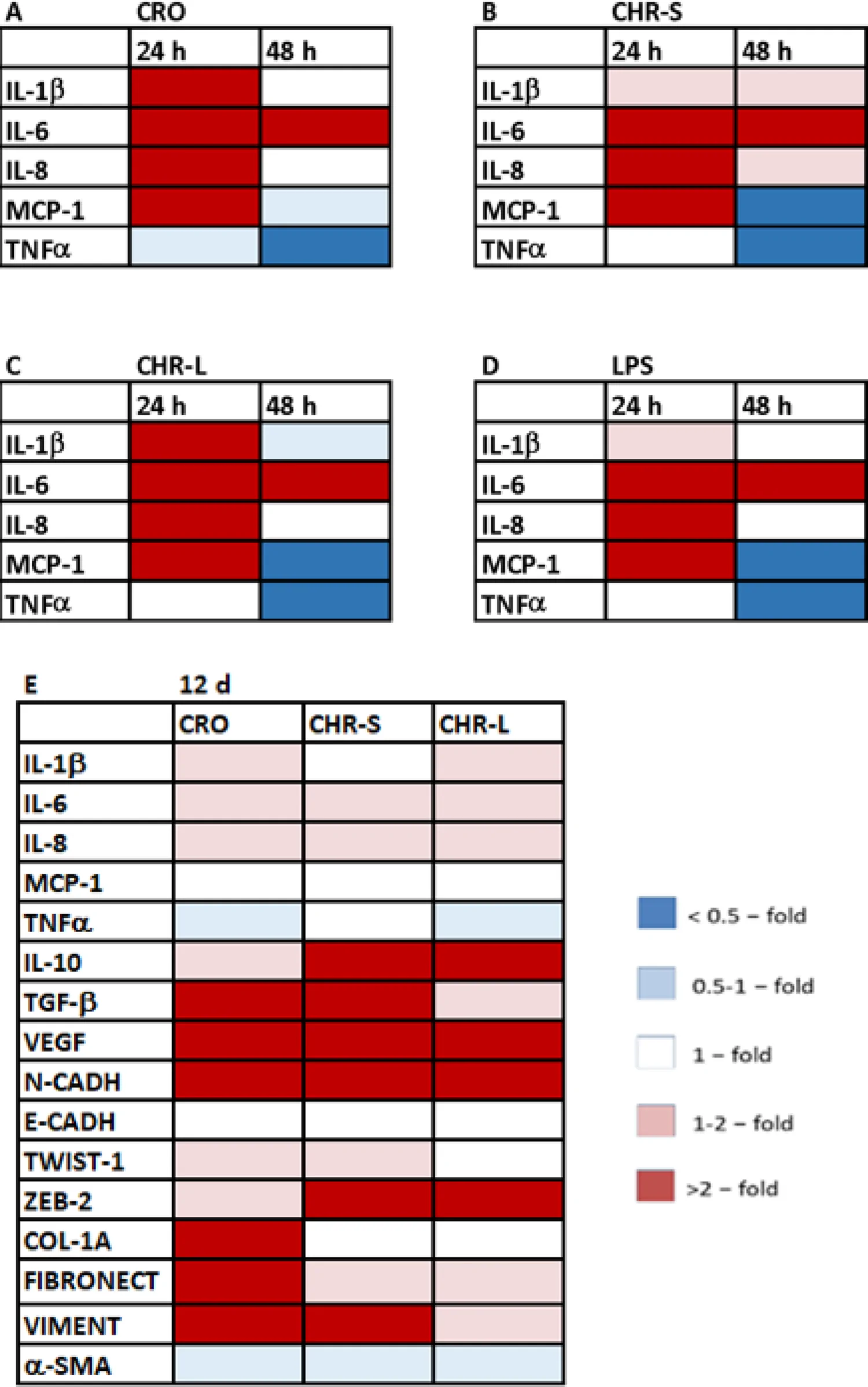
Expression of pro-inflammatory mediators in EpiAirway™ tissues. Gene expression analysis by qPCR after exposure to 100 µg/mL mineral fibres for 24–48 h (**A**, **B**, **C** and **D**) and 12 d (**E**). Data are expressed as fold change relative to PBS-treated vehicle controls and normalized to the HPRT-1 housekeeping gene. Statistical analysis was performed using one-way ANOVA followed by Tukey’s multiple comparison test, comparing each fibre-treated group to the corresponding vehicle control at the same time point. Significant differences (*p* < 0.05) are indicated by coloured rectangles

### Gene expression profile

The inhalation of airborne asbestos is well-known for inducing a persistent state of inflammation in the lung tissue, contributing to the insurgence of various pulmonary diseases. The inflammatory response induced by different asbestos fibres was evaluated in the EpiAirway™ tissues by analysing the gene expression profile of several pro-inflammatory mediators and their release in the extracellular milieu.

The gene expression profile (i.e., IL-1β, IL-6 IL-8, MCP-1, and TNFα analysis) was performed by qPCR after 24, 48 h, and 12 d of single-dose fibre exposure (100 µg/ml) (Fig. 5A–C). LPS (5 µg/ml) was used as a positive control to induce an early pro-inflammatory response after 48 h (Fig. 5D).

The cytokine expression profile revealed a uniform response of EpiAirway™ tissues to all stimuli during the first 48 h. In detail, exposure to CRO, CHR S, and CHR L fibres induced a significant upregulation of IL-1β, IL-6, IL-8 and MCP-1 after 24 h (pink and red rectangles), similarly to LPS. However, after 48 h, the transcriptional profile of inflammatory mediators returned to basal levels (white rectangles) or slightly decreased (blue rectangles), except for IL-6 which remained overexpressed in all treatments, and for IL-1β and IL-8 in CHR S-treated tissues (Fig. 5B).

Conversely, TNF-α gene expression remained unchanged after 24 h in the presence of CHR S, CHR L, and LPS, and slightly decreased with CRO, whereas it was significantly downregulated at 48 h in all treatments, including the LPS positive control.

After 12 d (Fig. 5E), fibre-treated tissues still showed a significant upregulation of IL-6 and IL-8, together with IL-1β (except in CHR S treatment for the latter), indicating a sustained production of inflammatory signals in the bronchial tissue even after a single exposure to asbestos fibres. Interestingly, the expression of IL-10 was also upregulated reaching ~ 2-fold increase with CRO, and > 2-fold increase with both CHR fibres, probably as a feedback response to the prolonged production of pro-inflammatory mediators. Moreover, 12 d-treated tissues were also analysed for changes in the expression profile of fibrosis and EMT markers. Specifically, both fibrosis markers TGF-β and Fibronectin were significantly upregulated with all three fibres, while Collagen-1 A was upregulated only in presence of CRO. α-SMA was slightly downregulated across all fibre treatments. Most EMT markers were significantly upregulated with all fibre treatments. In particular, the transcription factor ZEB-2 was upregulated in the presence of CRO (~ 2-fold increase) and strongly overexpressed with both CHR fibres (> 2-fold increase), while the transcription factor TWIST-1 was increased by CRO and CHR S. VEGF, N-Cadherin and Vimentin were significantly overexpressed in all fibre treatments. Conversely, expression of E-Cadherin, a typical marker of epithelial cells, remained unchanged in all treatments. These results indicate the instauration of a chronic inflammatory condition in the bronchial tissue together with a significant pressure towards EMT in epithelial cells with the three asbestos fibres. Notably, no significant differences in the gene expression profile were observed between CHR S and CHR L treatment, other than slightly increased EMT marker values in CHR S compared to CHR L, indicating that both long and short chrysotile can induce chronic effects.

### Release of pro-inflammatory cytokines

**Fig. 6:**
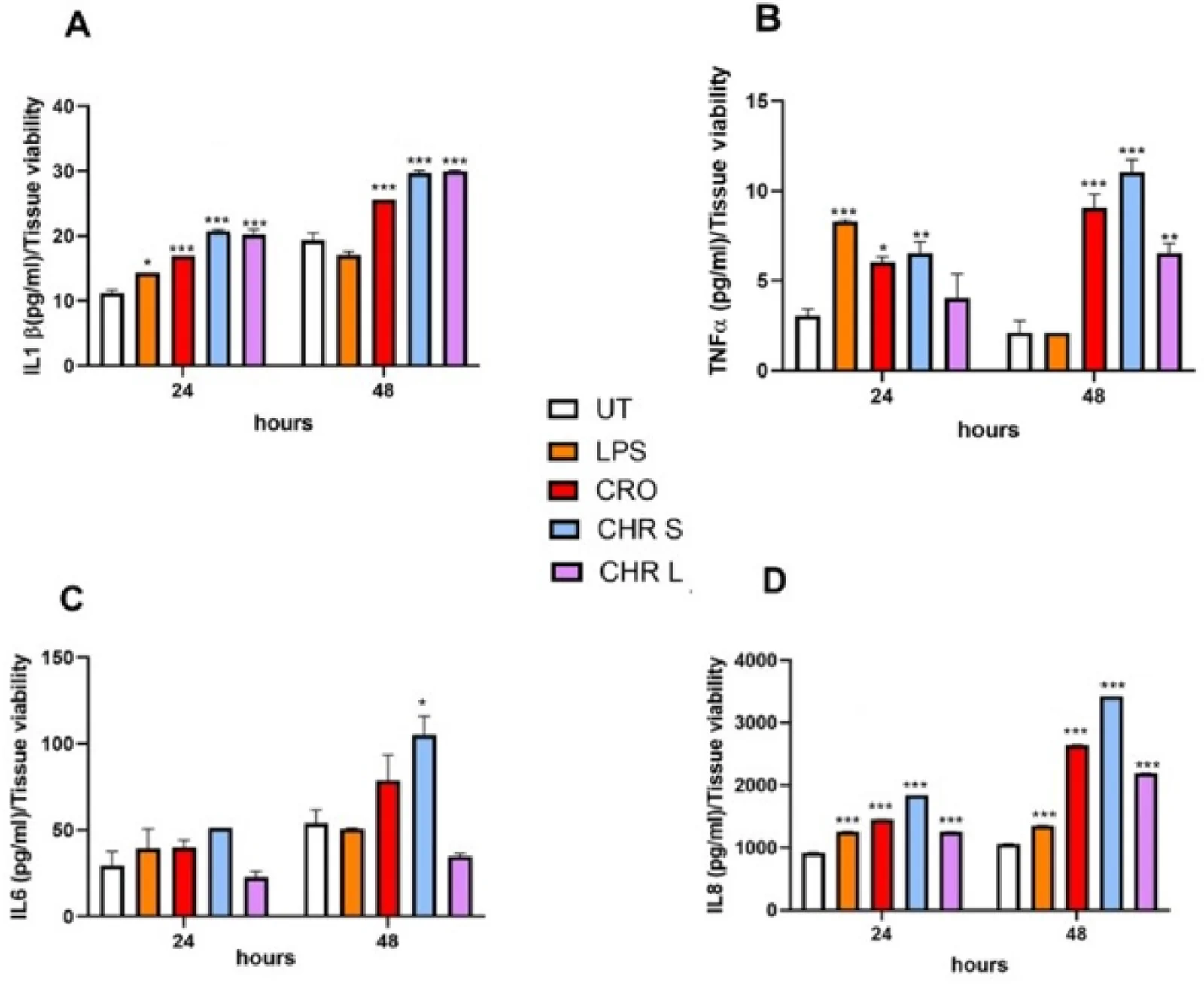
Release of pro-inflammatory mediators in the culture medium of EpiAirway™ tissues after 24 and 48 h exposure to 100 µg/mL mineral fibres. UT represents the untreated tissues. **A** IL-1β, **B** TNF-α, **C** IL-6 and **D** IL-8 concentrations were quantified using the MILLIPLEX^®^ Human Cytokine/Chemokine/Growth Factor Panel A. Cytokine concentrations (pg/ml) were normalized to MTT–derived metabolic activity at the corresponding time points in each treatment to account for differences in tissue viability. Data represent the mean ± SD of three independent biological experiments, each performed with two technical replicates that were averaged prior to statistical analysis. Error bars represent the standard deviation of biological replicates. Statistical analysis was performed on normalized values using two-way ANOVA followed by Bonferroni’s post-test. Comparisons were made between each treatment and the corresponding UT control at the same time point. *p < 0.05, **p < 0.01, ***p < 0.001 vs. UT

The release of several inflammatory mediators was measured into the culture media of fibre- and LPS-treated EpiAirway™ tissues after 24 and 48 h of treatment (Fig. 6A–D). IL-1β release was significantly increased in all fibre-treated tissues at 24 h, reaching even higher values at 48 h (Fig. 6A) compared to untreated control tissues (UT). In both CHR-treated samples, IL-1β release was significantly higher compared to CRO-treated tissues, with no significant differences between CHR S and CHR L. Conversely, LPS induced a significant release of IL-1β only at 24 h.

TNF-α protein release (Fig. 6B) was significantly increased in the media of all fibre-treated tissues at both timepoints, with an evident time-dependent increase compared to the negative control. Values were similar for all fibre-treated tissues at 24 h, however, at 48 h, the release in CHR L- treated tissues was markedly lower respect to CRO- and CHR S- treated tissues. LPS treatment increased TNF-α release only at 24 h compared to the negative control.

Regarding IL-6 (Fig. 6C), no significant increase in its secretion was measured at 24 h. Interestingly, at 48 h, IL-6 release was significantly elevated in CHR S-treated tissues, as compared to all other treatments and to the negative control.

Finally, IL-8 release raised significantly in response to all the three fibre treatments in a time-dependent manner (Fig. 6D). At both timepoints, all tested asbestos fibres triggered higher IL-8 release than LPS, with CHR S consistently promoting the greatest secretion of this chemokine in the culture media. LPS also induced a significant increase in IL-8 compared to untreated control tissues, with values remaining relatively stable over time.

**Fig. 7:**
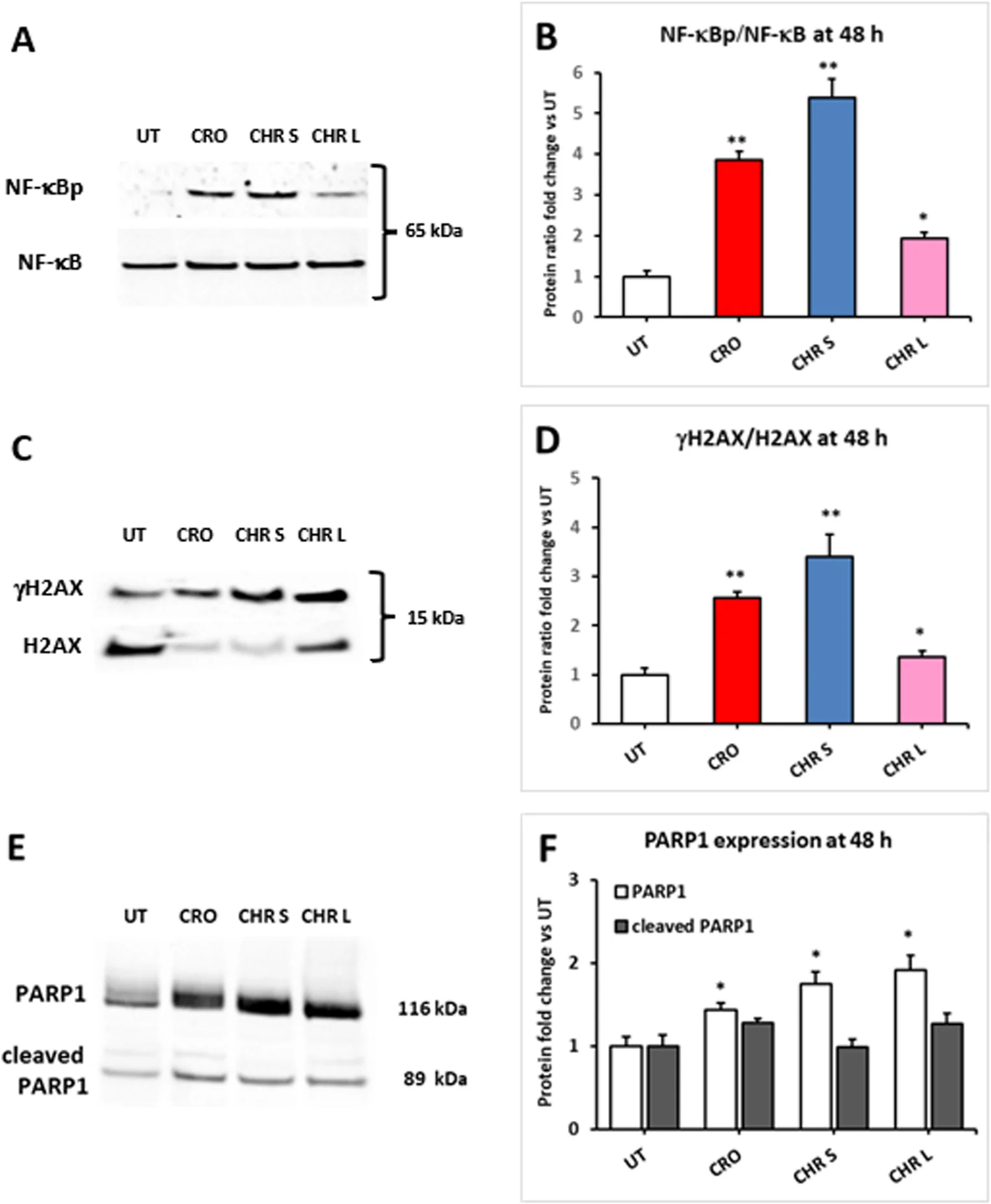
Western blot analyses of protein markers in EpiAirway tissues after 48 h of CRO, CHR S and CHR L (100 µg/mL) fibre treatments. **A** phospho-NF-κB(p65) and NF-κB(p65) protein expression immunoblot in untreated (UT) and fibre-treated tissues**. B** The bar chart shows the ratio of NF-κB(p65)p/NF-κB(p65) for each treatment, and all values are normalized on the total protein normalization (TPN method) of the correspondent lane on the SDS PAGE. Values are expressed as fold change compared to untreated cells (UT) and represent the mean ± SD of two separated experiments run in duplicate. *p < 0.05 and **p < 0.01 vs. UT (One-way ANOVA followed by Bonferroni post-test). **C** γ-H2AX and H2AX protein expression as signal of genotoxic damage, immunoblot of phosphorylated and not-phosphorylated H2AX, respectively. **D** The bar chart shows the ratio of γH2AX/H2AX expression calculated as reported for chart **B**. *p < 0.05 and **p < 0.01 vs. UT. **E** PARP1 and cleaved PARP1 protein expression immunoblot. **F** The bar chart shows PARP1 and cleaved PARP1 expression as fold change compared to UT and normalized by TPN. *p < 0.05 vs. UT

**Fig. 8:**
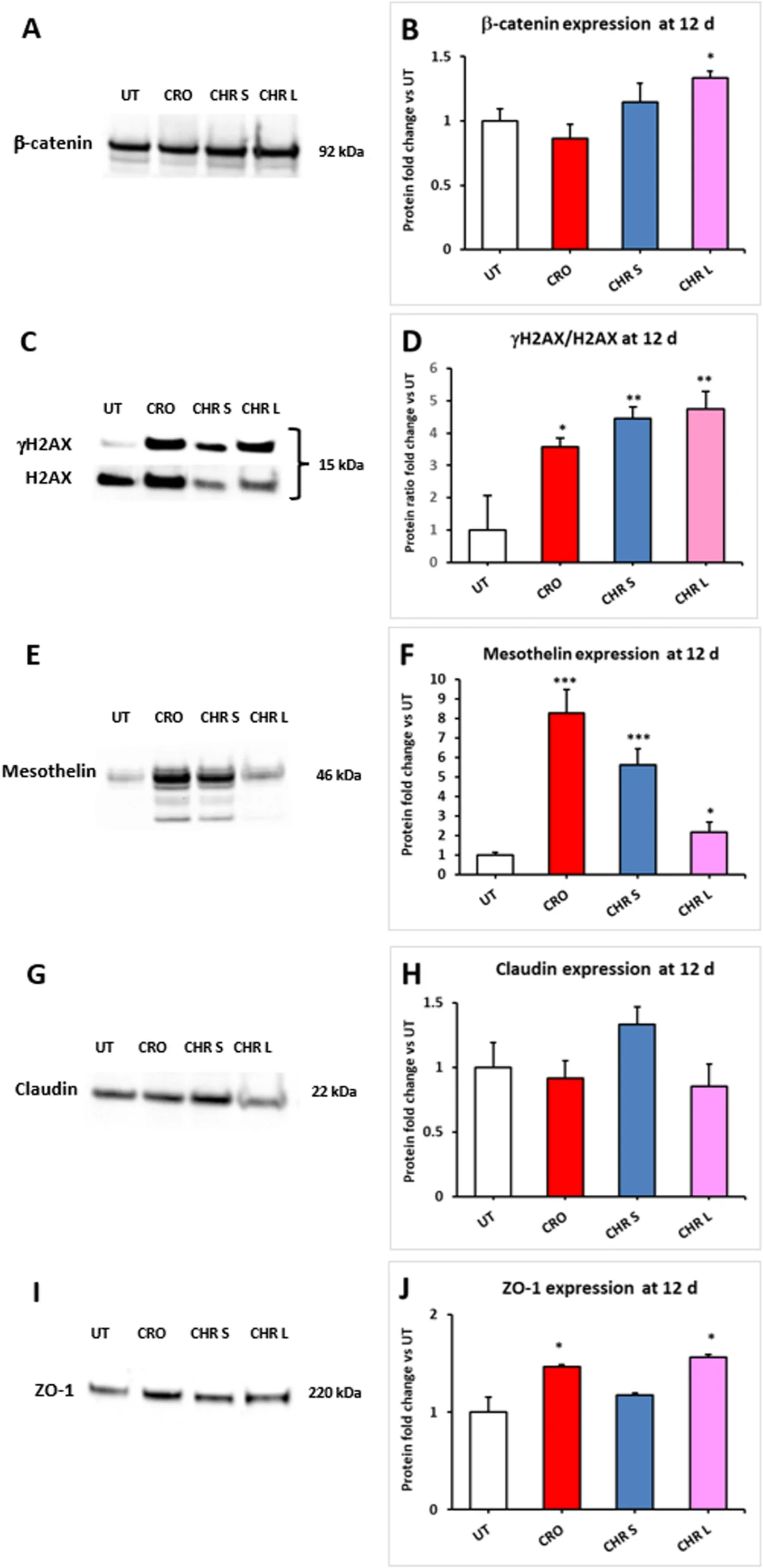
Western blot analyses of protein markers in EpiAirway tissues after 12 d of CRO, CHR S and CHR L (100 µg/mL) fibre treatments. **A** β-catenin protein expression immunoblot. **B** The bar chart shows β-catenin expression as fold change compared to untreated cells (UT) and normalized by the total protein normalization (TPN) method. Values represent the mean ± SD of two separated experiments run in duplicate. *p < 0.05 vs. UT (One-way ANOVA followed by Bonferroni post-test). **C** γ-H2AX and H2AX protein expression levels. **D** The bar chart shows the ratio of γH2AX/H2AX expression calculated as reported for chart **B**. *p < 0.05 and **p < 0.01 vs. UT. **E** Mesothelin protein expression immunoblot. **F** The bar chart shows Mesothelin expression as fold change compared to UT and normalized by TPN. *p < 0.05 and ***p < 0.001 vs. UT. **G** Claudin protein expression immunoblot. **H** The bar chart shows the Claudin expression as fold change compared to UT and normalized by TPN. **I** ZO-1 protein expression immunoblot. **J** The bar chart shows the ZO-1 expression as fold change compared to UT and normalized by TPN. *p < 0.05 vs. UT

### Analysis of protein expression and DNA damage

Protein expression in EpiAirway™ tissues was evaluated by Western blot analysis (WB) to confirm engagement of inflammatory master transcription factors (TF), as well as of proteins involved in DNA damage repair, apoptosis, EMT, and long-term cell transformation.

In addition to qPCR, the inflammatory response was further investigated by measuring NF-κB TF activation through phosphorylation (NF-κBp) after 48 h fibre exposure (Fig. 7A, B). NF-κBp is the active form of the transcription factor, translocating into the nucleus and binding specific DNA consensus sequences of pro-inflammatory mediators (Liu et al. 2017). All fibres promoted a significant increase of NF-κBp compared to untreated tissues. The highest increase was found in CHR S-treated tissues (> 5-fold increase), followed by CRO- (> 3-fold increase) and finally CHR L- (~ 2-fold increase) relative to the negative control.

Fibre-induced genotoxic damage was assessed as expression of phosphorylated histone protein H2AX, i.e. γ-H2AX (Fig. 7C, D), a well-known marker of DNA double strand breaks (DSBs) (Kopp et al. 2019). γ-H2AX levels were significantly increased in all fibre-exposed EpiAirway™ tissues compared to untreated controls, with CHR S showing the strongest induction (> 3-fold increase), followed by CRO (> 2-fold increase) and CHR L (~ 1.4-fold increase). Genotoxic stress was also investigated through analysis of PARP1 protein, a key enzyme involved in the nuclear DNA repair process of single- and double-strand breaks (Ko and Ren 2012), whose cleaved form is also considered a hallmark of apoptosis (Mashimo et al. 2021). Thus, both the native (116 kDa) and the cleaved (89 kDa) PARP1 expression levels were quantified in fibre-exposed tissues as compared to the untreated control (Fig. 7E, F). The native PARP1, indicating the active DNA repair enzyme (panel **F**, white bars), resulted significantly increased after 48 h in all fibre-treated tissues, ranging from 1.4-fold increase in CRO- to 1.8-fold in CHR S- and 1.9-fold in CHR L-exposed tissues as compared to controls. Conversely, the cleaved PARP1 form (panel **F**, grey bars) did not show significant changes under any condition, suggesting no marked induction of apoptosis at this time point.

WB analyses of EMT markers and of DNA damage persistence were subsequently performed in tissues treated for 12 d, as shown in Fig. 8.

First, we analysed expression levels of β-catenin, a well-recognized TF whose overexpression is critically involved in tumorigenesis and cancer progression (Fodde and Brabletz 2007). A significant increase was shown only in CHR L-treated tissues, (~ 1.4-fold vs. controls), whereas no changes were detected with the other fibre types (Fig. 8A, B). To monitor persistence of DNA damage after long-term exposure, γ-H2AX expression was re-evaluated in 12 d-treated tissues. γ-H2AX remained significantly elevated in all fibre-treated samples compared to the untreated control and, notably, reached higher levels than those observed at 48 h (Fig. 8C, D). In particular, the highest expression was found in CHR L-treated samples (~ 4.8-fold increase) followed by CHR S (~ 4.4-fold increase), and CRO (~ 3.6-fold increase).

We also analysed the release of 8-oxo-deoxy-Guanosine (8-oxo-dG), an oxidized DNA base adduct and established marker of DNA damage, in the tissue culture media at 7 and 12 d of mineral fibre treatment (Fig. 1S). Surprisingly, no significant increase was detected at both time points in fibre-treated tissue culture media compared to the negative control, indicating that, although the DNA DSBs were clearly visible in the tissues by γ-H2AX accumulation, oxidized DNA adducts were not detectable in the extracellular media at the same time points.

Finally, we focused on the expression of typical EMT markers, whose dysregulation is known to promote cell invasion consequent to loss of epithelial polarization (Kyuno et al. 2021; He et al. 2017), such as the well-known tumour antigen Mesothelin, and tight junction components Zonula Occludens (ZO-1) and Claudin-1. The cleaved form of Mesothelin (46 kDa), physiologically expressed on the plasma membrane of mesothelial cells but critically overexpressed in different cancers like pleural mesothelioma and lung adenocarcinoma (He et al. 2017), was significantly increased in EpiAirway™ tissues with all fibre treatments after 12 d. The induction was particularly marked in CRO- (8.2-fold) and CHR S-treated tissues (5.6-fold), whereas CHR L caused a moderate increase (2.1-fold). (Fig. 8E, F). Then, we assessed the levels of Claudin-1 tight junctional protein, since it demonstrates a highly variable expression in cancer by either promoting or inhibiting EMT depending on the type of tumour (Kyuno et al. 2021). In the EpiAirway™ model, the expression of this protein did not show any significant change after a prolonged treatment with the different asbestos fibres (Fig. 8G, H**)**. Conversely, the ZO-1 factor, whose overexpression together with β-catenin has been reported to promote EMT in many cancers (Polette et al. 2007), was found upregulated by ca. 1.5-fold after 12 d-exposure in o CRO- and CHR L-treated tissues compared to the untreated control and to CHR S (Fig. 8I, J).

## Discussion

To date the in vitro toxicity of respirable mineral fibres has been mainly assessed by 2D monoculture or simple co-culture cell models, which only partially mimic the complexity of the human lung epithelium. In recent years, several types of commercially available 3D in vitro human lung epithelial models have been developed, offering a more physiological representation of the distinct regions of the respiratory tracts (Barkauskas et al. 2017). In particular, the EpiAirway™ model consists of primary Normal Human Tracheobronchial Epithelial cells isolated from non-smoking patients. This 3D airway construct has been approved by the European Centre for the Validation of Alternative Methods (ECVAM) for the safety assessment of inhalable compounds (Pavlovska et al. 2021).

To fill the knowledge gap about the mechanisms occurring in the lung environment and initiating long-term fibrosis/EMT processes in the first days of exposure to asbestos fibres, the EpiAirway™ model can provide a reliable in vitro platform to monitor the progression of these phenomena. Furthermore, this human organotypic model could be used to assess early toxicological predictive markers to foresee the pathogenicity of highly heterogeneous mineral fibres.

Within the framework of the Adverse Outcome Pathway (AOP) for mineral fibre inhalation, our findings contribute to defining key events occurring downstream of the molecular initiating event (MIE), namely fibre deposition and interaction with epithelial cells. Early key events observed in this study include NF-κB activation, acute pro-inflammatory cytokine upregulation, and induction of DNA damage markers (γ-H2AX and PARP1). These were followed by persistent DNA damage, sustained inflammatory signalling, and upregulation of EMT- and fibrosis-related markers after prolonged exposure (12 days), which may represent later key events associated with tissue remodelling and potential progression toward carcinogenesis. Framing the results in this context helps distinguish transient early stress responses from sustained alterations that may lead to chronic pathological outcomes.

This approach contributes to the reconstruction of specific AOPs for fibre inhalation and supports the development of reliable and urgently needed in vitro toxicological tests. To complement mechanistic in vitro studies, the use of the Fibre Potential Toxicity Index (FTPI) model has been recently proposed (Gualtieri et al. 2024). The model aims at evaluating how physical-chemical mineral fibre parameters prompt adverse effects in vivo leading to carcinogenesis, by linking these parameters to the 10 IARC KCs (Smith et al. 2016). FPTI is a quantitative predictive model of toxicity/carcinogenicity of mineral fibres based on 18 physical-chemical and morphological parameters. The model delivers an index for mineral fibres to classify their potential toxicity and carcinogenicity. It was shown how the 18 FPTI parameters of the mineral fibres that induce specific adverse effects are tied to pathological processes attributed to the 10 IARC KCs (Smith et al. 2016; Gualtieri et al. 2024). Therefore, an optimized approach for mineral fibre predictive toxicology could combine the FPTI model with advanced in vitro toxicological tests investigating selected KCs previously identified as early distinctive biosignatures of fibre-induced malignancies in the lung.

To reach this aim, the toxic mechanisms of a Russian chrysotile (CHR), and of crocidolite UICC, widely used as a positive carcinogenic fibre control, were investigated using the EpiAirway™.

The potential toxicity of short chrysotile fibres (≤ 5 μm in length) remains widely debated due to the contradictory results reported in the literature (Gualtieri et al. 2023 and references herein). Thus, CHR fibres were divided into two different size fractions: CHR S, ≤ 5 μm length, and CHR L, > 5 μm length, to investigate the mechanisms of CHR toxicity linked to the fibre length parameter.

Initial MTT tests showed a transient and comparable cytotoxic effect in bronchial tissues with all fibre exposure (i.e. et 24–48 h, Fig. 2) with respect to untreated controls. This effect does not appear to reflect actual cell loss, as TEER values and histological analyses showed maintenance of the epithelial barrier integrity and of the tissue thickness at those time points (see Figs. 3 and 4). Furthermore, no evidence of apoptosis was detected in the treated tissues at 48 h as indicated by absence of cleaved PARP1 protein expression (Fig. 7E). Thus, the MTT results more likely reflect a transient impairment of the mitochondrial function, with acute cellular suffering immediately after fibre exposure, rather than significant cell death. This is further supported by the results at 12 d, where no cellular damage was observable (both at single or double dose exposure), indicating a functional recovery of the fibre-exposed tissues, which would be unlikely if the 24–48 h toxicity values (> 40% reduction respect to control) reflected a real decrease in cell number. This is in contrast with what observed in 2D human cell models, showing markedly affected macrophages and endothelial cells exposed to the same asbestos fibres up to 7 d (Mirata et al. 2025). The observed differences may result from inherent variations in cell-type sensitivity. Furthermore, the 3D tissue models show a resilience and functional recovery more consistent with in vivo responses (Keshavan et al. 2023), supporting its utility as a predictive tool for in vitro lung toxicological studies. TEER values can be used as a marker of overall tissue integrity (Srinivasan et al. 2015) reflecting tight junction (TJ) dynamics in the EpiAirway™ cell barriers (Rotoli et al. 2020). In fibre-exposed EpiAirway™, no significant variations were observed between all fibre-treated and untreated samples up to 12 d (Fig. 3), regardless of single or double dose exposure. These findings indicate a resilience of the bronchial tissue during the acute phase of fibre treatment, probably supported by the presence of basal cells that continuously regenerate the epithelium (Mistry et al. 2020). Consistent with the TEER results, the histological analyses, widely employed as diagnostic markers of tissue toxicity, showed overall maintenance of tissue integrity (Fig. 4). However, qualitative differences in the tissue morphologies could be observed in asbestos-treated EpiAirway™ tissues compared to the controls at 12 d. In particular, H&E and Alcian Blue staining highlighted a higher number of mucous-secreting cells in all fibre-treated tissues, while Sirius Red indicated increased new collagen synthesis and deposition in CHR S-, and more so, in CRO-exposed tissues. In the latter, new collagen production was also supported by the overexpression of Col-1 A gene after 12 d (Fig. 5E). Overall, the MTT, TEER and histological analyses indicate that, although no evidence of tissue damage resulted after 12 d, fibre exposure induced increased mucus production and collagen deposition, reflecting the early bronchial responses observed in vivo (Raby et al. 2023; Kayalar et al. 2024). However, it should be acknowledged that the EpiAirway™ model lacks immune and stromal components, such as macrophages and fibroblasts, which are central mediators of inflammation and fibrosis in vivo. Therefore, while this system is valuable for dissecting epithelial-driven mechanisms, extrapolation of the results to the complex pulmonary environment should be performed with caution. Finally, although these data capture some initial bronchial reactions, the lack of distinctive alterations currently limits the use of these markers as early predictors of carcinogenesis.

Other important sentinels of the onset of many severe diseases, including cancer, are inflammatory mediators. It is well known that inflammation is an early response to tissue injury, and it plays a fundamental role in the tissue repair success or failure as well as for disease chronicization, fibrosis, and cancer development, especially in the lung, an organ continuously exposed to external noxious stimuli (Kayalar et al. 2024). Both in vitro and in vivo studies should also consider the fundamental issue of the time frame choice used to evaluate critical predictive carcinogenic endpoints, such as inflammation. As highlighted by Poland and collaborators (2024), especially when considering inflammation as a predictive marker, it is important to distinguish between an initial generic inflammatory process, which may represent a normal, protective reaction to all noxious stimuli, and prolonged adverse effects leading to chronic inflammation and contributing to cancer development. Selecting appropriate experimental timing is essential to reliably differentiate between fibres causing a transient inflammatory response from those inducing a persistent, pathological inflammation. This would finally allow the correct use of inflammatory markers as reliable carcinogenic predictors also in this context. In our study the experimental times were set at 48 h and 12 d to be able to measure an early, as well as a prolonged in vitro inflammatory response, with the aim to capture the initiation of chronicization.

TNF-α and IL-1 are known to increase in asbestos-exposed animals and play central roles in the inflammatory response, together with IL-6 and IL-8, and in the subsequent development of fibrosis (Acencio et al. 2015). In all EpiAirway™ fibre-treated tissues, a significant acute response (i.e. during the first 24–48 h) in terms of cytokine release and/or overexpression was observed (Figs. 5 and 6), with no substantial differences among the three asbestos types. Notably, IL-1β, IL-6 and IL-8, whose prolonged overexpression is linked to chronic inflammation and lung cancer development (Poland et al. 2024; Wu et al. 2025), remained elevated even after 12 d treatment, despite the absence of immune cells in the EpiAirway™ that would normally amplify inflammatory responses. These results indicate that bronchial epithelial cells alone may sustain a persistent inflammatory signalling in response to the initial injury caused by the mineral fibres. This could represent the onset of a chronic process, as immune cells would be subsequently recruited, although resulting unable to eliminate the biodurable cause of the damage. Thus, the prolonged inflammatory signals observed in the EpiAirway™ fibre-treated tissues up to 12 d could serve as an early prognostic KC of mineral fibre toxicity/carcinogenicity in predictive in vitro toxicology tests.

The master TF driving pro-inflammatory responses in immune cells, NF-κB (Liu et al. 2017), was activated in tissues exposed to asbestos. NF-κBp expression can be prompted by the persistence of ROS in the intracellular environment, and their production has been extensively documented in asbestos-treated cell models (Mirata et al. 2022, 2025). As also mentioned by Manning et al. (2002), ROS represent the critical link between asbestos fibres and asbestos-related diseases, through the production of pro-inflammatory mediators stimulated by NF-κBp.

Another fundamental sign of toxicity, which could have dramatic consequences for cell transformation and could be used as a predictive carcinogenic KC, is DNA damage. In asbestos-treated EpiAirway™, this was demonstrated by the overexpression of DNA-repair enzyme PARP1 at 48 h, and by the persistent overexpression of γH2AX from 48 h up to 12 d treatments (Figs. 7 and 8), with no significant differences among the three asbestos fibres. γH2AX long-term overexpression appears particularly promising as a prognostic marker of mineral fibre carcinogenicity. In contrast, quantification of 8-oxo-dG in the EpiAirway™ culture media did not show significant accumulation over time as compared to the control tissues (Fig. 1S). These results may indicate that asbestos fibres induce DNA DSBs in the absence of DNA oxidation, or alternatively, that oxidative DNA damage occurs, but oxidized base adducts are not sufficient for quantification in the tissue media at the investigated time points. At 12 d, 8-oxo-dG does not appear as a reliable early predictive marker of fibre toxicity in EpiAirway™. Conversely, the presence of sustained γH2AX foci from 48 h to 12 d, occurring in the absence of cell death, supports the persistence of DNA DSBs as an early toxicity predictive marker. Given that DNA DSBs are well-recognized drivers of cell transformation favouring carcinogenic mutations and chromosomal translocations (Lechner et al. 1985a, b), their detection may provide mechanistically relevant insight into fibre-induced transformation processes. In recent past, other 2D bronchial epithelial models have reported asbestos-induced DNA damage and epigenetic alterations, including DNA methylation changes (Gulino et al. 2016; Emerce et al. 2019), suggesting that early genomic instability and epigenetic dysregulation are central events in fibre-driven carcinogenesis.

Our findings are consistent with these observations in that persistent genotoxic stress (γH2AX positivity) and sustained inflammatory signalling were also detected in the 3D bronchial model. However, unlike many 2D systems where cytotoxicity and oxidative stress often dominate early endpoints, the EpiAirway™ tissues exhibited functional recovery and preserved barrier integrity despite the activation of transformation-related pathways. This difference may reflect the higher structural and functional complexity of 3D tissues, which better reproduce epithelial organization, tight junction dynamics, and regenerative capacity.

Another important marker of cancer initiation and progression is the β-catenin TF overexpression, responsible for the enhanced transcription of well-known oncogenes such as c-Myc and cyclin D-1 (Shang et al. 2017). However, in fibre-treated EpiAirway™ at 12 d, this TF was only found slightly increased in CHR L-treated samples (Fig. 8) suggesting that this time point may be too early and not appropriate to highlight the β-catenin-driven carcinogenic pathway in bronchial tissue.

If maintained over time, early molecular alterations could evolve into transformation-associated pathways, including EMT activation, a crucial step for the onset of chronic lung diseases and cancer development like MPM and lung carcinoma (Ramundo et al. 2021). At 12 d, the presence of numerous fibrotic and EMT signals was clearly and significantly detected in all fibre treatments in EpiAirway™ as compared to controls (Figs. 5E and 8), including increased expression of specific growth factors (i.e. TGF-β, and VEGF), EMT-driving TF (i.e. TWIST-1, and ZEB-2), and well-known EMT expression markers (i.e. ZO-1, Fibronectin, N-Cadherin, Vimentin, and Mesothelin). Among the three asbestos fibres, CRO seemed to induce the highest EMT signals as compared to CHR S and L, which in turn elicited comparable responses among them and significantly higher than controls.

Particularly interesting as potential fibre predictive KC markers were the overexpressed N-Cadherin, Vimentin and Mesothelin detected already at 12 d, since these proteins play an important role in EMT progression, which is crucial to tumorigenesis (Loh et al. 2019; Kyuno et al. 2020; Ramundo et al. 2021).

N-Cadherin is a well-known indicator of ongoing EMT for its involvement in cell migration and proliferation; it is linked to β-catenin pathway activation and to ZO-1 expression regulation, both increased in some of the asbestos-treated tissues (Fig. 8). Its overexpression has been correlated with the development of various types of carcinomas (Hulit et al. 2007; Hui et al. 2013). Vimentin, a filamentous cytoskeletal protein, is considered a canonical EMT biomarker and is consistently overexpressed in metastatic cancer cells where it contributes to stemness, plasticity, and resistance to stress (Usman et al. 2021). Finally, Mesothelin is a glycoprotein usually undetected in normal tissues, except for mesothelial cells of the peritoneal and pleural cavities; it is involved in the promotion of metastasis and in the regulation of cancer growth, as well as of EMT, and it is a recognised tumour metastatic diagnostic marker (He et al. 2017). These proteins, which are routinely used as markers of established and frequently metastatic tumours, were found to be overexpressed at 12 d in the asbestos-treated EpiAirway™. These findings suggest that EMT-related processes could already begin in the in vitro model after a relatively short period of fibre contact, compared with the prolonged latency typically observed in vivo after years from the first harmful inhalation. Therefore, these markers represent promising candidates as early KC markers in predictive toxicology tests of mineral fibres.

In the EpiAirway™ model, no major differences were observed between CHR S and L harmful effects, apart from a slightly higher expression of EMT markers in CHR S-treated tissues compared to CHR L. This finding may suggest a comparable carcinogenic potential between short and long chrysotile fibres, as also observed in other studies (Gualtieri et al. 2023; Mirata et al. 2025), although this topic still arises controversies. Indeed, if DNA damage and EMT phenomena can be already observed at 12 d of tissue/fibre contact, it means that the onset of cellular transformation mechanisms may initiate right after the first inhalation. Thus, attention should be paid to short chrysotile fibres. In fact, our data indicate that a few weeks may be sufficient for early molecular events associated with cell transformation induced by chrysotile short fibres.

**Table 3:** Quantification of possible early carcinogenic markers

Marker	CRO		CHR S		CHR L	
	48 h	12 d	48 h	12 d	48 h	12 d
Cytotoxicity	2	–	2	–	2	–
TEER Alteration	–	–	–	–	–	–
Histological changes	–	3	–	2	–	2
Genotoxicity	3	3	3	3	2	3
Inflammation	12	6	13	5	11	6
EMT	n.d.	22	n.d.	14	n.d.	12

Summary of cell/tissue alterations, related to cell transformation, as possible early markers of mineral fibre carcinogenicity in the EpiAirway™ model. Values in the cells indicate increased alteration as compared to untreated control (UT). The values in each cell were obtained as the Ʃ of values attributed to each quantified marker. For cytotoxicity (MTT), TEER, and histological changes (Ʃ of H&E, Alcian Blue, Sirius Red), 1 point (pt) was assigned for differences up to 25%; 2 pt for differences between 25 and 50%; 3 pt for differences over 50%, compared to UT, respectively. For markers of genotoxicity (Ʃ γ-H2AX, 8-oxo-dG quantification), inflammation (Ʃ IL1β, IL6, IL8, MCP1, TNF-α expression) and EMT (Ʃ TGFβ, VEGF, Mesothelin, Vimentin, N-Cadherin, TWIST-1, ZEB-2, Fibronectin, Collagen-1 A, ZO-1, α-SMA expression), 1 pt was assigned for an increase up to 1.5 fold; 2 pt for increases between 1.5 and 3.0 fold; 3 pt for increases over 3.0 fold; -1 pt for decrease up to 0.5 fold; -2 for decreases over 0.5 fold, compared to UT, respectively. Hyphens indicate no change respect to UT; n.d., not determined. All calculations for each fibre treatment are detailed in Supplementary file 2

## Conclusions

The aim of this study was to recapitulate in vitro, in a human 3D bronchial model, the early alterations caused in the lung by exposure to the carcinogenic crocidolite and long and short chrysotile asbestos fibres, and to possibly identify early KC markers for the setup of predictive toxicology tests for mineral fibres. All parameters and time points investigated are summarized and quantified in Table 3, for each mineral fibre. The early signs observed in bronchial tissues at 48 h, namely toxicity (cyto- and geno-), slight histological changes, and acute inflammation, represent generic responses, as they are typical of any acute tissue damage, and hardly exclusively associated with carcinogenesis. Thus, these alterations, but especially this early time point, are not suitable for defining specific and reliable KC markers of mineral fibre carcinogenicity in vitro. Conversely, after 12 d of asbestos fibre exposure, in the EpiAirway™ a number of marked alterations attributable to the IARC KCs could indicate the onset, already at this time point, of cell transformation events, possibly leading to cancer development. Based on these findings, we propose that the following sentinel alterations may serve as relevant KC endpoints in 3D in vitro predictive models of mineral fibre carcinogenicity, assessed at a minimum exposure duration of approximately 12 d: (i) a marked genotoxicity, in the absence of actual cell death signals, monitored by persistence of nuclear γH2AX positivity, a recognized and reliable biomarker for DNA DSBs and genotoxicity assessment, already proposed by Kopp et al. (2019) (ii) Sustained inflammatory signalling, indicative of chronicization in the tissue, monitored by IL-1β, IL-6 and IL-8 overexpression analysis, and (iii) a significant increase of crucial fibrosis/EMT markers, such as, TGF-β, VEGF, ZEB-2, N-Cadherin, Vimentin and Mesothelin, possibly highlighted by both gene and protein expression analyses.

These alterations correspond to the IARC KCs 2 (genotoxicity), 6 (chronic inflammation), and 8 (cell immortalization/proliferative signalling and related transformation processes) respectively. Their quantitative determination following 12 d-fibre treatment in 3D lung in vitro models may provide mechanistically relevant evidence that adverse transformation pathways have been initiated within a relatively short experimental timeframe, potentially reflecting processes that, in vivo, evolve over prolonged exposure periods and may contribute to cancer development. Finally, we propose that a comprehensive model for evaluating mineral fibre carcinogenicity should integrate the assessment of these three KC-based biosignatures in 3D lung models at approximately two weeks of exposure, together with the preliminary screening by predictive tools, such as the FPTI model. This approach would enable the scientific community to define the carcinogenic potential of any respirable fibre, and to support the implementation of suitable precaution measures for exposed individuals.

## Supplementary Information

Below is the link to the electronic supplementary material.

Supplementary Material 1

Supplementary Material 2

## Data Availability

Included in the paper or Supplementary Information.
