# Supplementary material for: Defining early carcinogenic markers for the prediction of the hazard potential of mineral fibres in an in vitro human 3D bronchial model
Source: Arch Toxicol. 2026 Jun 15;100(10):4777–97. doi: 10.1007/s00204-026-04471-3 (PMC13593725; doi:10.1007/s00204-026-04471-3)
Supplement: Supplementary file 1 [file 204_2026_4471_MOESM1_ESM.docx]

**Defining early carcinogenic markers for the prediction of the hazard potential of mineral fibres in an in vitro human bronchial 3D model**

Vanessa Almonti ^a^, Serena Mirata ^b,c^, Mario Passalacqua ^c,d^, Stefania Vernazza ^c,d^, Sara Tirendi ^c,d^, Sara Ferrando ^b,e^, Beatrice Risso ^b^, Elena Grasselli ^b,c,f,g^, Giulia De Negri Atanasio ^b,c^, Anna Maria Bassi ^c,d^, Alessandro Francesco Gualtieri ^h^, Sonia Scarfì ^b,c,e,*^

^a^ Wielkopolska Center for Advanced Technologies, Adam Mickiewicz University, Poznan, 61-614, Poland

^b^Department of Earth, Environment and Life Sciences, University of Genova, 16132 Genova, Italy.

^c^ Inter-University Center for the Promotion of the 3Rs Principles in Teaching & Research (Centro 3R), 56122 Pisa, Italy.

^d^ Department of Experimental Medicine, University of Genova, 16132 Genova, Italy.

^e^ National Biodiversity Future Center (NBFC), 90133 Palermo, Italy

^f^ National Center for the Development of New Technologies in Agriculture (Agritech), Napoli, Italy

^g^ Istituto Nazionale Biostrutture e Biosistemi (INBB), Roma 00136, Italy

^h^ Department of Chemical and Geological Sciences, University of Modena and Reggio Emilia, 41125 Modena, Italy.

**Materials and Methods**

1. **8-oxo-deoxy-Guanosine quantification in the EpiAirway™ tissue media**

The levels of 8-oxo-dG were analysed in the EpiAirway™ collected media after 7 and 12 d of fibre treatment, i.e. after a single 100 µg/ml dose exposure and compared to untreated controls. The oxidized DNA base adduct was quantified by the specific 8-OHdG ELISA Kit (ThermoFisher, Milan) following the manufacturer’s instructions. In brief, the medium collected during the experiment was measured with a competitive enzyme immunoassay. The optical density of each treatment group at 450 nm was recorded.

Supplementary Figure 1 (Fig. 1S)

**Fig. 1S: 8-oxo-deoxyGuanosine quantification in tissue culture media.** The DNA oxidized base adduct accumulation was determined by ELISA assay in the EpiAiraway™ tissue media after treatment with 100 mg/ml mineral fibres at 7 and 14 d (gray and black bars, respectively) and compared to the release from untreated tissues (UT) at the same time points. Values are expressed as ng/ml and are the mean of 2 independent experiments run in duplicate.

Supplementary Figure 2 (Fig.2S)

**Fig. 2S: 8-oxo-deoxyGuanosine calibration curve.** Calibration curve of the 8-oxo-dG ELISA Kit. Values are expressed as ng/ml and are the mean of 3 replicates.
